# A comprehensive review of monkeypox virus and mpox characteristics

**DOI:** 10.3389/fcimb.2024.1360586

**Published:** 2024-03-06

**Authors:** Emmanuel Alakunle, Daniel Kolawole, Diana Diaz-Cánova, Faith Alele, Oyelola Adegboye, Ugo Moens, Malachy Ifeanyi Okeke

**Affiliations:** ^1^ Department of Natural and Environmental Sciences, American University of Nigeria, Yola, Nigeria; ^2^ Department of Medical Biology, UIT – The Arctic University of Norway, Tromsø, Norway; ^3^ School of Health, University of the Sunshine Coast, Sippy Downs, QLD, Australia; ^4^ Menzies School of Health Research, Charles Darwin University, Darwin, NT, Australia

**Keywords:** monkeypox, genomics, evolution, antivirals, epidemiology, infection biology, biosafety, one health

## Abstract

Monkeypox virus (MPXV) is the etiological agent of monkeypox (mpox), a zoonotic disease. MPXV is endemic in the forested regions of West and Central Africa, but the virus has recently spread globally, causing outbreaks in multiple non-endemic countries. In this paper, we review the characteristics of the virus, including its ecology, genomics, infection biology, and evolution. We estimate by phylogenomic molecular clock that the B.1 lineage responsible for the 2022 mpox outbreaks has been in circulation since 2016. We interrogate the host-virus interactions that modulate the virus infection biology, signal transduction, pathogenesis, and host immune responses. We highlight the changing pathophysiology and epidemiology of MPXV and summarize recent advances in the prevention and treatment of mpox. In addition, this review identifies knowledge gaps with respect to the virus and the disease, suggests future research directions to address the knowledge gaps, and proposes a One Health approach as an effective strategy to prevent current and future epidemics of mpox.

## 1 Introduction

*Monkeypox virus* (MPXV) is the etiological agent of a zoonotic disease called monkeypox (mpox). It is a double-stranded DNA (dsDNA) virus belonging to *Orthopoxvirus* (OPXV) genus within the *Poxviridae* family and *Chordopoxvirinae* as the subfamily (Alakunle et al., 2020). Other members of this genus include *Variola virus* (VARV), *Cowpox virus* (CPXV), *Vaccinia virus* (VACV), *Camelpox virus* (CMLV), *Taterapox virus* (TATV) and *Ectromelia virus* (ECTV). MPXV is divided into Clade I and Clade II, with Clade II subclassified as Clade IIa and IIb (Happi et al., 2022). For five decades, MPXV was endemic in West and Central Africa (Earl et al., 2012), and exportation of the virus to non-endemic regions was rare (Alakunle and Okeke, 2022). However, the incidence (since 2017) of mpox outside endemic regions has increased, and the epidemiological profile of the disease within endemic regions has changed (Grothe et al., 2022). This may have led to the MPXV emergence and re-emergence in endemic countries in 2022 (Alakunle and Okeke, 2022). This paper will cover the current state of knowledge on the characteristics of MPXV and mpox, the infection biology, molecular pathogenesis, and evolution of MPXV as well as the clinical features, diagnosis, epidemiology, and therapeutic options against mpox. In addition, the review will critically interrogate and evaluate the contributions of viral, host, and anthropogenic factors to the emergence and reemergence of mpox across the globe.

Before 1970, there was no documented report of human MPXV infection, although the virus had previously caused infections in monkeys and apes (Arita and Henderson, 1968). Infections in monkeys were reported in laboratory/captive animals and were first identified in captive monkeys in Denmark in 1958. The first human mpox case emerged in a 9-month-old boy in the Democratic Republic of the Congo (DRC) in August 1970 (Ladnyj et al., 1972). Subsequently, six additional mpox cases were identified between September 1970 and April 1971 in Liberia, Sierra Leone and Nigeria (Lourie et al., 1972). Since then, MPXV has been reported in several countries and is endemic in Benin, Cameroon, the Central African Republic, the DRC, Gabon, Ivory Coast, Liberia, Nigeria, the Republic of the Congo, Sierra Leone, and South Sudan (Bass et al., 2013; World Health Organization, 2022a).

**Figure 1:**
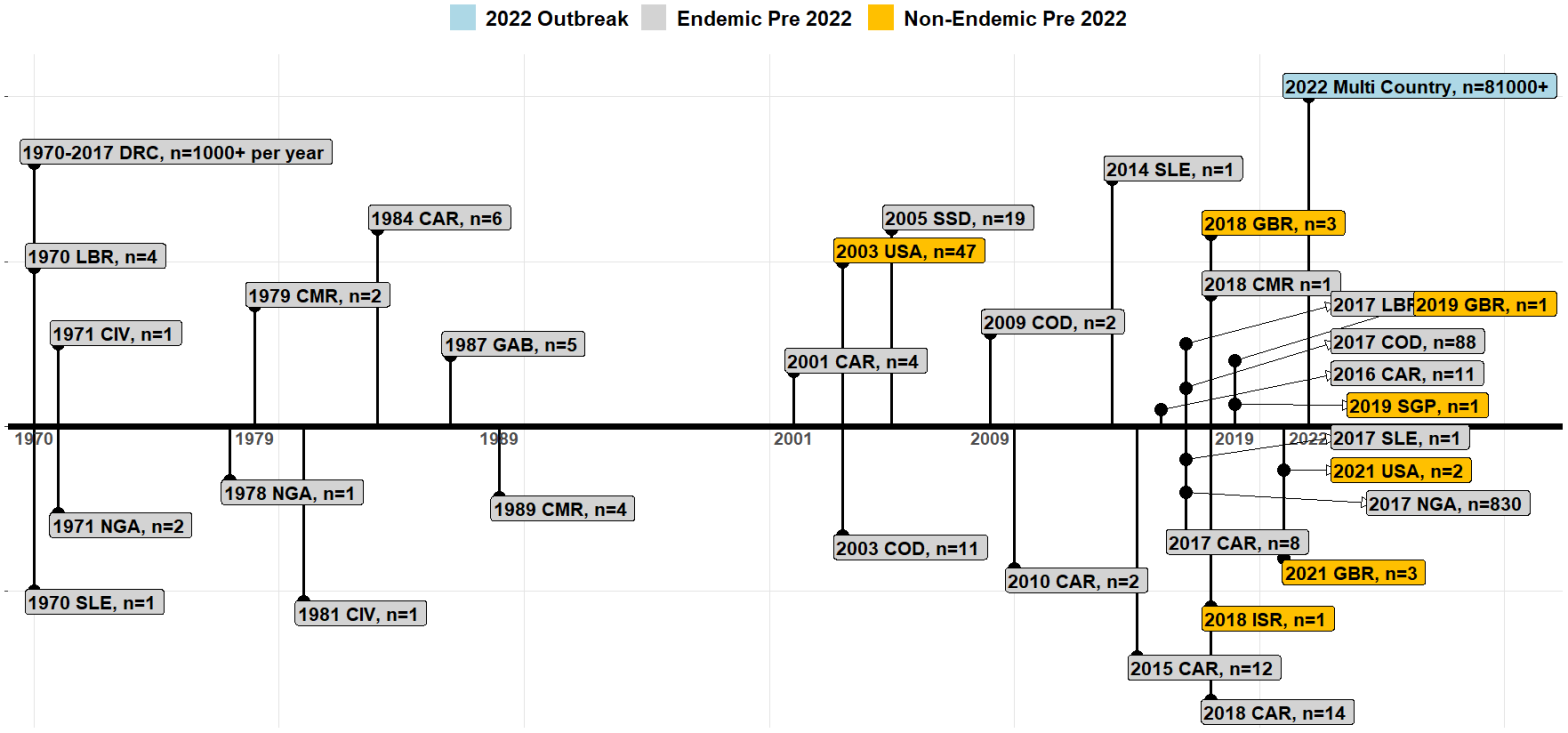
Timeline of MPXV emergence and re-emergence in endemic regions and globally. Each event timeline indicates the pre-2022 outbreak in endemic and non-endemic countries and the 2022 mpox outbreak (Durski et al., 2018; World Health Organization, 2022). Note: Cameroon (CMR), the Central African Republic (CAR), Cote d’Ivoire (CIV), the Democratic Republic of the Congo (DRC), Gabon (GAB), the United Kingdom (GBR), Liberia (LBR), Nigeria (NGA), Israel (ISR), Sierra Leone (SLE), Singapore (SGP), the Republic of the Congo (COD), South Sudan (SSD), the United States of America (USA).

**Figure 1** displays the global mpox outbreak timeline. Between 1970 and 2021, the cases have been sporadic and geographically limited within endemic regions (Brown and Leggat, 2016; Titanji et al., 2022). Notably, the DRC is the only country that continuously reports yearly cases of mpox with tropical rainforest regions accounting for 98.7% of all cases pre-2022 (Brown and Leggat, 2016; Durski et al., 2018). In Nigeria, sporadic cases were reported in the 1970s; however, re-emergence of the disease started in 2017 with an eleven-year-old boy as the index case in Bayelsa state (Yinka-Ogunleye et al., 2018). At the end of 2017, Nigeria recorded 88 cases, and during this outbreak, travel-related cases in non-endemic countries were reported, including the United Kingdom (UK), the United States of America (USA), Israel, and Singapore, between 2018 and 2021 (Adegboye et al., 2022).

The first mpox outbreak in a non-endemic country was reported in 2003 in the USA linked to importation of rodents from Ghana (**Figure 1**) (Anderson et al., 2003; Centre for Disease Control, 2003; Croft et al., 2007). By the end of the outbreak, 47 people had been infected (10 probable and 37 confirmed cases) (Centre for Disease Control, 2003; Center for Diseases Control and Prevention (CDC), 2023). There were no other travel-related cases reported until 2018. Between 2018 to 2021, 11 travel-related mpox cases were recorded in the UK, Singapore, Israel, and the USA (**Figure 1**). Of these, four resulted in secondary cases: one healthcare worker in the UK was infected by contaminated bedding, an adult and a child from a family from the UK had a travel history to Nigeria, and one traveler to Israel who had visited Nigeria in 2018. Between 2019 and 2021, a total of seven mpox outbreaks occurred outside Africa in Singapore, the UK and the USA (**Figure 1**). All travel related cases originated in Nigeria, with high-throughput sequencing confirming it as Clade II (Vaughan et al., 2018; Erez et al., 2019; Fang et al., 2020; Hobson et al., 2021; Bunge et al., 2022). Between 2017 and October 30, 2022, a total of 830 cases were recorded in 33 out of 36 states in Nigeria (Nigeria Centre for Disease Control (NCDC), 2022).

**Figure 2:**
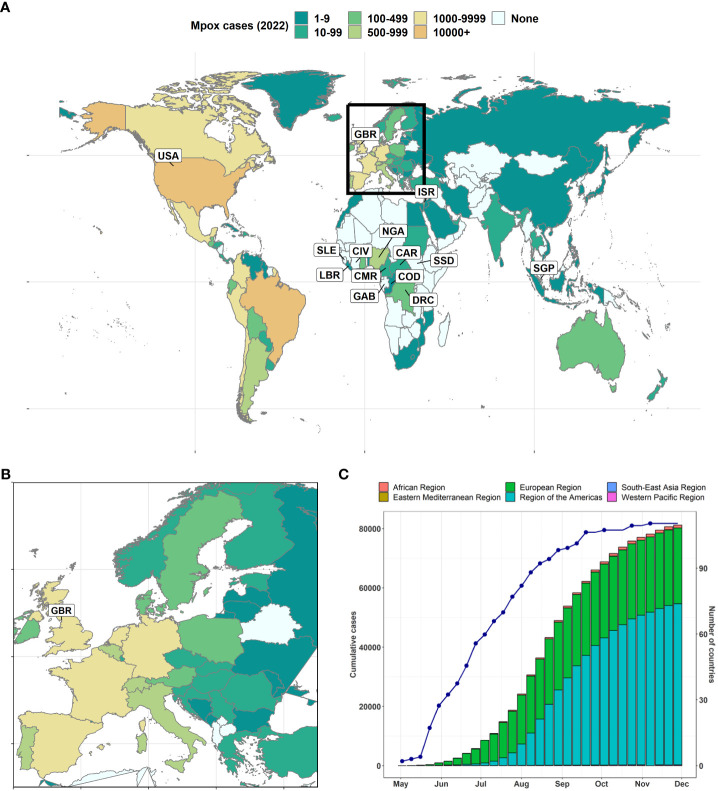
Timeline of MPXV re-emergence and global spread. **(A)** Global map of 2022 mpox of the geographical distribution of the outbreak as of December 5, 2022 (Durski et al., 2018; World Health Organization, 2022). The names of countries with at least one case pre-2022 are labelled. **(B)** Global maps zoom on Europe. **(C)** Weekly cumulative number of cases reported to World Health Organization (WHO) stacked by WHO region. The line/dot represents the cumulative number of countries affected. Countries: Cameroon (CMR), the Central African Republic (CAR), Cote d’Ivoire (CIV), the Democratic Republic of the Congo (DRC), Gabon (GAB), the United Kingdom (GBR), Liberia (LBR), Nigeria (NGA), Israel (ISR), Sierra Leone (SLE), Singapore (SGP), the Republic of the Congo (COD), South Sudan (SSD), the United States of America (USA).

The current global mpox outbreak started in May 2022 (Alakunle et al., 2020; Adegboye et al., 2022) and was declared a public health emergency of international concern on July 23, 2022 (Nuzzo et al., 2022). As of August 02, 2023, there were a total of 88,600 laboratory-confirmed cases and 152 deaths (case-fatality rate, 0.17%) across 113 countries including 106 countries that have not historically reported mpox (**Figures 2A-C**) (World Health Organization, 2022). The Americas recorded the highest number of cases during the 2022 mpox outbreak, with the USA (n = 29,513) and Brazil (n = 10,168) accounting for 48.32% of the total cases (**Figure 2C**). Other notable affected countries include Spain (n = 7,408), France (n = 4,110), Colombia (n = 3,880), the UK (n = 3,730), Germany (n = 3,673), Peru (n = 3,561), Mexico (n = 3,455), and Canada (n = 1,459). In Africa, Nigeria has the highest mpox cases with 634 cases.

## 2 Ecology, host range, tissue and cell tropism

Despite the name, monkeypox, monkeys are not the genuine reservoir of MPXV. Several animals can naturally or experimentally be infected with MPXV (**Table 1**) (Li et al., 2023), but the natural host reservoir remains elusive. While specific host-cell receptors are responsible for cell tropism, the specificity of MPXV is yet to be determined. Factors like the monkeypox inhibitor of complement enzymes (MOPICE) and complement control protein (CCP) can influence the viral cellular and tissue tropism (Hudson et al., 2012). Nonetheless, a wide spectrum of tissue and host tropism is expected, which may explain the possibility of MPXV establishing animal reservoirs in non-endemic regions (Kmiec and Kirchhoff, 2022). Organs such as ovaries, kidneys, heart, brain, pancreas, liver, and lung have been identified as some of the tissue tropism for MPXV (Arthur et al., 2022). However, specific virus ligands remain unidentified. The inability to identify specific virus ligands and cognate host receptors for MPXV tropism suggests that the virus uses many alternative ligands to successfully invade host cells or the host receptors have functional redundancy to the virus ligand. Spillover to humans (zoonotic transmission) might arise from the disruptions of the natural habitats of wild animals (Domán et al., 2022). This could occur via various routes, including aerosol, direct contact, and fomite transmission (Walker, 2022). It is believed that the MPXV outbreaks in Africa prior to 2022 occurred as a result of a spillover from animals to humans (Faye et al., 2018; Kabuga and El Zowalaty, 2019; Petersen et al., 2019b; Happi et al., 2022; Riopelle et al., 2022). Thus, there is a likelihood of MPXV being sustained in the spillover due to the wide geographical coverage of the MPXV hosts (Tu, 2015).

**Table 1:** List of natural MPXV-infected animals and experimental MPXV-infected animals.

MPXV HOST RANGE & RESERVOIR HOSTS			
Natural MPXV-Infected Animals	References	Experimental MPXV-Infected Animals	References
Sooty mangabey monkey (*Cercocebusatys*)	(Alakunle et al., 2020)	Prairie Dog (*Cynomysludovicianus*)	(Parker and Buller, 2013; Domán et al., 2022)
Gambian-pouched rat (*Cricetomysgambianus*)	(Alakunle et al., 2020)	Mouse (BALB/c and C57BL/6)	(Parker and Buller, 2013; Domán et al., 2022)
Rhesus macaques (*Macacamulatta*)	(Alakunle et al., 2020)	Gambian Pouched Rat (*Cricetomysgambianus*)	(Parker and Buller, 2013)
Cynomolgus macaque (*Macacafascicularis*)	(Alakunle et al., 2020)	Crowned monkeys *(Cercopithecus ascanius)*	(Parker and Buller, 2013)
Asian Monkeys (*M. fascicularis*)	(Alakunle et al., 2020)	Red-tailed monkeys *(Cercopithecus pogonias)*	(Parker and Buller, 2013)
Southern opossum (*Didelphis marsupialis*)	(Alakunle et al., 2020)	White-nosed monkeys (*Cercopithecus petaurista*)	(Parker and Buller, 2013)
Sun squirrel (*Heliosciurus*sp.)	(Alakunle et al., 2020)	Western colobus monkey (*Colobus badius*)	(Parker and Buller, 2013)
African hedgehogs (*Atelerix*sp.)	(Alakunle et al., 2020)	Rhesus macaque (*Macacamulatta*)	(Parker and Buller, 2013)
Jerboas (*Jaculus*sp.)	(Alakunle et al., 2020)	Cynomolgus macaque (*Macacafascicularis*)	(Parker and Buller, 2013)
Woodchucks (*Marmota monax*)	(Alakunle et al., 2020)	Thomas’s rope squirrel *(Funisciurusanerythrus)*	(Parker and Buller, 2013)
Shot-tailed opossum (*Monodelphisdomestica*)	(Alakunle et al., 2020)	Red-legged sun squirrel (*Heliosciurusrufobrachium*)	(Parker and Buller, 2013)
Porcupines (*Atherurusafricanus*)	(Alakunle et al., 2020)	Ribboned rope squirrel (*Funisciuruslemniscatus*)	(Parker and Buller, 2013)
Giant anteaters (*Myrmecophagatridactyla*)	(Alakunle et al., 2020)	Gambian sun squirrel (*Heliosciurusgambianus*)	(Parker and Buller, 2013)
Prairie dogs (*Cynomys*spp.)	(Alakunle et al., 2020)	Eurasia red squirrels (*Sciurus vulgaris*)	(Parker and Buller, 2013)
Elephant shrew (*Petrodromustetradactylus*)	(Alakunle et al., 2020)	Thirteen-lined ground squirrel (*Spermophilustridecemlineatus*)	(Parker and Buller, 2013)
Domestic pig (*Susscrofa*)	(Alakunle et al., 2020)	Rabbits	(Parker and Buller, 2013)
Rope squirrel (*Funisciurus*sp.)	(Alakunle et al., 2020)	Mouse (CAST/EiJ strain)	(Parker and Buller, 2013)
African dormice (*Graphiurus*spp.)	(Alakunle et al., 2020)	Cotton rats (*Sigmodon* sp.)	(Parker and Buller, 2013)

## 3 Genomics, phylogenomics, phylodynamics, and evolution

### 3.1 MPXV genome and gene content

**Figure 3:**
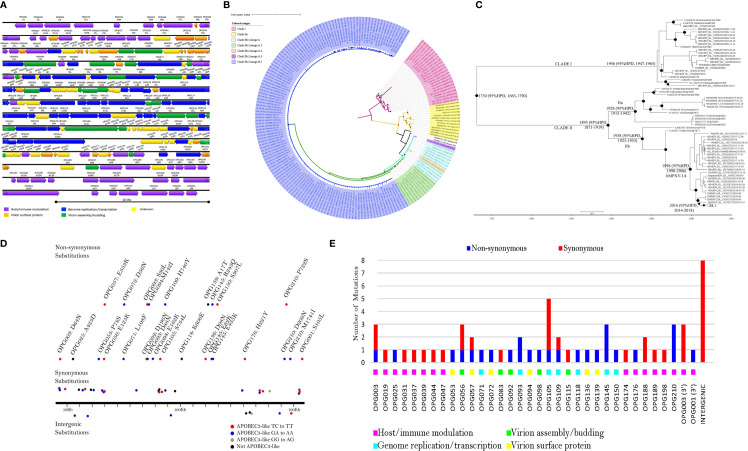
Genome annotation, phylogenomic tree, maximum clade credibility tree, and mutation map of MPXV. **(A)** Schematic presentation of MPXV genome. Annotation is given for MPXV reference strain NC_063383.1. The orientation of ORFs is given by the direction of arrow heads. ORFs are named according to the nomenclature of orthopoxvirus genes (example: OPG001) (Senkevich et al., 2021) and VACV Western Reserve nomenclature (example: J1L). MPXV genome consists of a conserved central region (OPG048 to OPG151) flanked by variable terminal regions, which contain inverted terminal repeats (ITR) (Shchelkunov et al., 2001; Shen-Gunther et al., 2023). The central region encodes genes for genome replication, essential enzymes, and structural proteins. Conversely, the variable terminal regions contain mainly virulence and host-range genes (Shchelkunov et al., 2001). MPXV genome encompasses >190 nonoverlapping open reading frames (ORFs) (Shchelkunov et al., 2001; Hendrickson et al., 2010; Shen-Gunther et al., 2023) and at least 4 ORFs are located in ITR (Shchelkunov et al., 2002; Likos et al., 2005; Nakazawa et al., 2013). ORFs are colored based on their function. **(B)** Bayesian Inference phylogenetic tree of concatenated 62 non-recombinant conserved genes from 197 MPXV genomes retrieved from GenBank and GISAID. Recombination detection program 4 (RDP4) (Martin et al., 2015) was used to detect recombination in the 62 conserved genes (Diaz-Cánova et al., 2022a) and the phylogenetic tree was reconstructed using MrBayes v3.2.7 (Ronquist et al., 2012), as previously published (Diaz-Cánova et al., 2022a). Black squares at the nodes indicate posterior probabilities ≥ 0.95. The scale bar represents expected substitutions per site. **(C)** Time-Scaled Bayesian Inference phylogenetic tree of concatenated 62 non-recombinant conserved genes from 197 MPXV strains. The presence of a temporal signal within the dataset was examined by regression of genetic divergence (root-to-tip genetic distance) and the sampling date using TempEst v.1.5.3 (Rambaut et al., 2016). The Maximum likelihood tree of 62 non-recombinant conserved genes built as described previously (Diaz-Cánova et al., 2022a) was used for TempEst. The maximum-clade-credibility (MCC) tree was generated using BEAST 1.10.4 (Suchard et al., 2018), using a log-normal strict clock, constant population size, and HKY substitution model. Markov Chain Monte Carlo (MCMC) chains were run until reaching convergence. The convergence of MCMC chains was checked by the effective sample size (ESS) values >200 for each parameter (after burn-in) using Tracer v1.7.1 (Rambaut et al., 2018). The maximum-clade-credibility (MCC) tree was generated using TreeAnnotator v1.10.4. Black circles at the nodes indicate posterior probabilities ≥ 0.9. The scale bar represents expected substitutions per site. **(D)** Mutation map showing all 60 consensus substitutions. 51 of the 60 consensus nucleotide substitutions possessing APOBEC3 like pattern of mutation (GA > AA, GG > AG, and TC > TT). Twenty-eight of these were GA > AA substitutions, two were GG > AG (this mutational pattern is a product of APOBEC3G), twenty-one were TC > TT, and the remaining nine substitutions were not typical of APOBEC3 editing. **(E)** Synonymous and non-synonymous mutational count.

MPXV has a long and complex genome of 196 Kbp - 211 Kbp with a conserved central region and variable inverted terminal repeats (ITR) (**Figure 3A**). Within MPXV clades, Clade I isolates have more uniform genome length (196 Kbp - 199 Kbp) than Clade II isolates (196 Kbp - 211 Kbp). The length of MXPV ITR varies between 6.5 Kbp to 17.5 Kbp (Shchelkunov et al., 2002; Likos et al., 2005; Nakazawa et al., 2013). Our genomic comparison of MPXV clades (Clade I, n=23; Clade IIa, n=13 and Clade IIb, n=161) indicated similar gene synteny (excluding the ITR), but slight differences in the gene content among the clades due to some genes that are missing or truncated in either one or two clades. The results are consistent with a previous study (Forni et al., 2022a) which indicates that four genes (*D14L, D15L, D16L* and *D17L*) are missing and three genes (*D4L, B14L* and *B15L*) are truncated in Clade II. Furthermore, the homologue of *VACV-Cop E5R* is only absent in Clade I and three genes (*K1R*, homologues of *VACV-Cop A47L* and *VACV-Cop B11R*) are truncated. Another major difference is the gene content of their left terminal in which Clade IIa contains four genes (*N3R, N2R, N1R* and *R1R*) that are absent in Clade I and IIb. However, these four genes are present on the right terminal of all clades. Furthermore, Clade I and IIb have the*D2L* gene. Within Clade IIb, gene content of the lineage A and B.1 are similar.

### 3.2 Phylogenomics and phylodynamics

Our Bayesian phylogenetic (BI) analysis of 62 non-recombinant conserved genes (Diaz-Cánova et al., 2022a) from 197 MPXV isolates resolved MPXV into three monophyletic clades, namely Clade I (Congo Basin Clade), Clade IIa (West Africa Clade) and Clade IIb consisting majorly of human MPXV (hMPXV) isolated between 2017-2022 (**Figure 3B**). Clade IIb was further divided into lineages: A (n=11), A.1 (n=11), A.2 (n=1), A.3 (n=1), and B.1 (n=135) as assigned by the GISAID (Global Initiative on Sharing All Influenza Data) (https://gisaid.org/). MPXV tree topology reported here is similar to that of the trees reported previously (Gigante et al., 2022; Isidro et al., 2022; Luna et al., 2022; Wang et al., 2022). Isidro et al. reported that the transition from A.1/A.1.1 to B.1 is characterized by a long, divergent branch (Isidro et al., 2022) which suggests accelerated microevolution. The findings of this current study (**Figure 3B**) agree with the suggestion.

Lineage A (hMPXV-1A) corresponds to the 2017–2019 outbreak, although it contains strains isolated after this time frame (**Figure 3B**) and this observation has been reported by others (Gigante et al., 2022). A recent study showed that new Nigerian hMPXV genomes isolated in 2019-2020 were identified as belonging to the lineage A (Ndodo et al., 2023). Lineage B.1 contains most hMPXV genomes from 2022 (**Figure 3B**). It is poorly resolved, and its sub-lineages cannot be unequivocally assigned (**Figure 3B**). The low clade supports are probably due to very low genetic variability among isolates (Scarpa et al., 2022). Polytomy within lineage B.1 could be an indication of uncertainty in the relatedness of isolates or the belief that those isolates evolved independently from a single origin (Slowinski, 2001; Phylogenetic pitchforks - Understanding Evolution, 2023), although recombination cannot be excluded (Yeh et al., 2022).

Furthermore, molecular dating analysis was carried out on the 62 non-recombinant conserved genes of 197 MPXV isolates to estimate the evolutionary rate and the time of the Most Recent Ancestor (tMRCA) (**Figure 3C**). TempEst analysis showed temporal signal in the dataset (R=0.65). Maximum Clade Credibility (MCC) tree (**Figure 3C**) demonstrated that MPXV emerged at 1730 (95% high posterior density interval (HPD), 1663 – 1790) with a mean evolutionary rate estimated to be 5.68 × 10^−6^ (subs/site/year), with 95% HPD of 4.53 × 10^−6^ − 6.86 × 10^−6^ subs/site/year. A recent publication estimated the substitution rate to be 5 x 10^-6^ (Dumonteil et al., 2023) which is in agreement with our result, but previous estimations of the substitution rates of Clades I and IIa were smaller (Gigante et al., 2022). Clades I, IIa, and IIb were estimated to have emerged in 1956, 1928, and 1938, respectively which is earlier by 33, 47, and 97 years as estimated by Forni et al. (Forni et al., 2022b). Lineage A and B.1 were estimated to have a tMRCA of 1998 (95% HPD, 1990 - 2006) and 2016 (95% HPD, 2014 – 2018), respectively. Our dating analysis put the emergence of the B.1 lineage six years earlier than that estimated by Luna et al. (Luna et al., 2022) and Nextclade (https://clades.nextstrain.org/). This discrepancy may be explained by molecular dating methods used (ML-TSP versus BI-MCC). We hypothesize that the emergence of lineage A about 1998 allowed enough time for lineage A evolution and that may explain the long divergent branch length from lineage A to B.1. Similarly, the emergence of lineage B.1 in 2016 in tandem with clustering of some 2022 isolates within the lineage A (2017-2019) support cryptic transmission of the B.1 viruses prior to the current 2022 outbreak in multiple non-endemic countries (Alakunle and Okeke, 2022; Dumonteil et al., 2023). A recent molecular clock analysis based on accumulation of APOBEC3 (apolipoprotein B mRNA editing enzyme, catalytic polypetide 3)-type mutations inferred that MPXV with APOBEC3 editing has been in circulation since at least 2016 (O’Toole et al., 2023), and this is in agreement with our molecular dating reported herein although their interpretation differs from ours. While we infer that B.1 lineage emerged in 2016, O`Toole et al. concluded that APOBEC3-type mutations, a predictor of human-to-human transmission emerged in 2016. The close phylogenetic relationship between A.1 isolates from Nigeria (in particular EPI_ISL_15370077) with B.1 suggests a single origin for lineage B.1.

### 3.3 Mutational analysis and recombination

Our mutational analysis revealed that lineage B.1 has 66-86 nucleotide substitutions (60 consensus) and 28-39 amino acid substitutions (26 consensus) compared to reference genome NC_063383.1, MPXV from 2018 (**Figures 3D, E**). Consensus substitutions predominantly affect genes responsible for host/immune modulation and viral replication/transcription (**Figure 3E**). *B21R* (*OPG210*) codes for a T cell suppressor; our analysis demonstrated that this open reading frame (ORF) contained three consensus amino acid substitutions (D209N, P722S, and M1741I). *A18R* (*OPG145*) contained three substitutions (E62K, R243Q, and E435K). Excluding *G8R* (*OPG093*) with two substitutions, all the other affected ORFs contained one consensus amino acid substitution each (**Figure 3E**). The impact of these substitutions on the pathogenicity, transmissibility, immune evasion, and host specificity of MPXV remains unknown. The E353K substitution within *F13L* (*OPG057*) affects the target for the antiviral agent, tecovirimat; however, functional studies have not revealed any effect on the efficacy of the drug (Gigante et al., 2022). Wang et al. also reported three mutations in OPG210 protein in MPXV 2022 outbreak strains (Wang et al., 2022). Additionally, they showed that this protein together with other nine proteins (D2L‐like, OPG023, OPG047, OPG071, OPG105, OPG109, A27L‐like, OPG153, and OPG188 proteins) have more mutations compared to the other MPXV proteins (Wang et al., 2022).

In contrast to our study, previous mutational studies have reported fewer consensus substitutions (Isidro et al., 2022; Wang et al., 2022; Wassenaar et al., 2022). They reported 46 shared mutations in MPXV genomes from 2022 outbreak compared with NC_063383 (Chen et al., 2005; Isidro et al., 2022). However the number of mutations identified here and in other studies is higher than one would expect for MPXV (Chen et al., 2005; Isidro et al., 2022; Ndodo et al., 2023), based on the low substitution rate of OPXV (Firth et al., 2010). The accelerated evolution of MPXV has been attributed to the human APOBEC3 (Isidro et al., 2022; O’Toole and Rambaut, 2022; Wang et al., 2022). Since most mutations identified here (**Figure 3D**) and elsewhere were GA>AA and TC>TT mutations, which are compatible with APOBEC3 activity. APOBEC3 is a cytidine deaminase known to play important functions in innate anti-viral immunity (Harris and Dudley, 2015; Salter et al., 2016). Evidence for the accumulation of APOBEC3 enriched substitutions in MPXV isolates has been reported since 2017, which corresponds to the detection of lineage A (Gigante et al., 2022). Ever since, every year, there has been an increase in APOBEC3-like mutations in MPXV (Ndodo et al., 2023). This mutational pattern has become more pronounced as suggested from more recent isolates (B.1) (Gigante et al., 2022; Ndodo et al., 2023), (**Figure 3D**). The recent pattern of upsurge of APOBEC3 derived mutations may be an indication of a change in virus-host interaction such as sustained human-human transmission (Ndodo et al., 2023) or a new route of infection (Gigante et al., 2022). Gigante et al. (Gigante et al., 2022) suggested the possibility of recombination in MPXV following observation of three sequences that showed a chimeric pattern in their genomes, although assembly errors could be a plausible explanation. Recently, tandem repeats and linkage disequilibrium analysis provided evidence of natural recombination in lineage B.1 (Yeh et al., 2022).

## 4 Infection biology and pathophysiology

### 4.1 Virus morphogenesis, pathogenesis and pathophysiology

**Figure 4:**
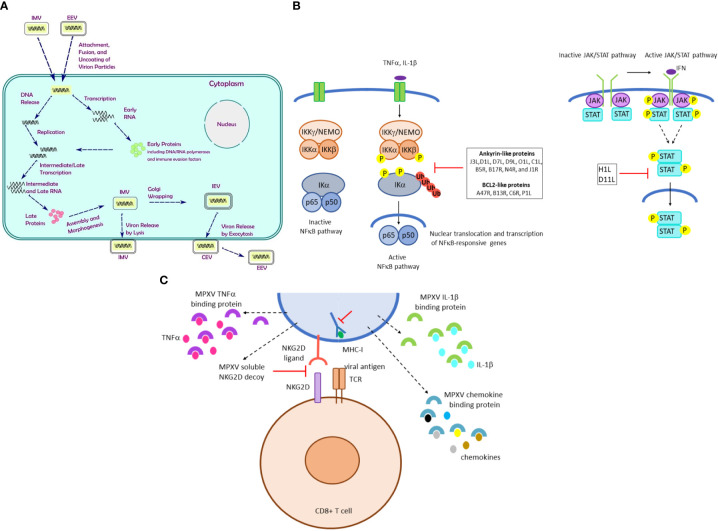
MPXV morphogenesis, signaling and immune evasion strategies. **(A)** The MPXV replication cycle. After attachment of the virion to the host cell membrane, the viral genome is released by uncoating followed by transcription and duplication of the viral genome. Translation and subsequent assembly results in IMV. MPXV virions can exist as intracellular mature virus (IMV), cell associated enveloped virus (CEV) and extracellular-enveloped virus (EEV). IMV assembles in the cytoplasm and consists of a core particle wrapped in a membrane. IMV particles egress from the infected cells by lysis, whereas some IMVs are transported through microtubules and wrapped by an intracellular membrane to produce an intracellular enveloped virus (IEV), which can further fuse with the cell membrane and be released to form EEV (Schmidt and Mercer, 2012; Sivan et al., 2016; Realegeno et al., 2020). Some EEVs remain attached to the cell surface (CEV) and are responsible for cell-to-cell spread, whereas EEV that detaches from the infected cells play a role in long-range dissemination within the host (Blasco and Moss, 1992). IMV particles are thought to be the form responsible for inter-host viral transmission. In contrast, EEV are known to be important for intra-host viral dissemination (Payne, 1980). **(B)** MPXV strategies to activate signaling pathways. Left panel: The canonical NFκB pathway requires activation of the cytoplasmic p65/p50 dimer, which is anchored into the cytoplasm through its interaction with IKα. The trimer IKKα/IKKβ/IKKγ will upon activation phosphorylate IKα, which is subsequently ubiquitinated and probed for proteasomal degradation. This results in release of p65/p50, which translocate to the nucleus and can induce transcription of NFκB target genes. Several MPXV proteins can perturb the NFκB pathway. Right panel: The JAK/STAT pathway consist of the tyrosine kinases JAK that phosphorylate and activate the transcription factor STAT. The MPXV proteins encoded by the genes *H1L* and *D11L* can inhibit activation of the JAK/STAT pathway. **(C)** MPXV avoids detection of virus-infection cells by cytotoxic CD8+T cells. Infected host cells will present viral peptide fragments by MHC-I molecules. These will be recognized by T cell receptors (TCR). Co-recognition of NKG2D ligand on the MPXV-infected cell and the NKG2D receptor on the CD8+ T cell is required. MPXV will prevent the latter interaction by expressing soluble NKG2D. Moreover, the cytokine gradient produced upon MPXV infection will be disturbed by MPXV-encoded proteins that will bind these cytokines such as TNFα, IL-1β.

Poxvirus mature particles are ovoid or brick-shaped with surface tubules and have a characteristic dumbbell-shaped nucleoprotein core containing the viral genome (Shchelkunov et al., 2001). MPXV virions are ~200 nm in diameter and ~300 nm in length (Wenner et al., 1968). Like other OPXVs, MPXV forms three distinct infectious virus particles: intracellular mature virus (IMV), cell associated enveloped virus (CEV) and extracellular enveloped virus (EEV) although CEV and EEV are morphologically and structurally indistinguishable. Morphogenesis and transmission of IMV, CEV, and EEV are described in **Figure 4A**.

Observations of cynomolgus monkeys infected with MPXV gave the first indications of the pathogenic role of this virus with intramuscular injection of the virus leading to an intense inflammatory immune response (Wenner et al., 1968, 1969). The pathogenesis of MPXV has also been studied in other animals, including rabbits, rodents and prairie dogs (Hutson et al., 2009; Bunge et al., 2022). Studies in monkeys, mice, and prairie dogs demonstrated that Clade I viruses are more virulent than Clade II strains, which reflects the situation in humans (Saijo et al., 2009; Hutson et al., 2010; Bunge et al., 2022; Americo et al., 2023; Falendysz et al., 2023). Except for disease severity, the clinical features of the two clades are similar (Kipkorir et al., 2022).

MPXV infection has an incubation period of 5–21 days and the most common symptoms for the 2022 human mpox outbreak in non-endemic regions based on 48,622 patients were skin lesions (95%), fever (58%), lymphadenopathy (53%), fatigue/asthenia (39%), myalgia (31%), and headache (30%) (Liu et al., 2023). Regarding skin lesions, anogenital lesions were most frequent (66%), followed by lesions on the trunk/torso (48%), face/head (39%), and extremities (~30%) (Liu et al., 2023). This is in contrast with previous human mpox outbreaks from 1980-2022 where skin lesions were most common on the face and extremities (Pourriyahi et al., 2023). Lymphadenopathy, which typically occurs 1-2 days before rash, is a distinct feature of MPXV which is used to distinguish it from smallpox and chickenpox (Altindis et al., 2022; McCarthy, 2022). The morphological progression of the rash is macular, popular, vesicular, and pustular lesions. The crust formed by pustules desquamate after 1-2 weeks (Altindis et al., 2022; McCarthy, 2022; Nakhaie et al., 2022). In the current outbreak, inguinal lymphadenopathy was more frequent than cervical and axillary lymphadenopathy (Liu et al., 2023), whereas in previous outbreaks in endemic countries submandibular, cervical and axillary lymphadenopathy were more frequent (Damon, 2011). The differences in clinical symptoms between human mpox infections before 2022 and the current outbreak are probably the result of different virus strain (Clade I versus Clade II, respectively) and patient group (both female and male youngsters versus mainly adult men having sex with men). In men having sex with men (MSM), atypical clinical symptoms such as genital lesions and anal ulcers were observed (Mitjà et al., 2022; Bragazzi et al., 2023).

MPXV can enter the host via the oral/respiratory tract, infecting the oral and respiratory tract mucosae, with the upper, middle and lower airway epithelium as main targets for primary infection (Giulio and Eckburg, 2004; Damon, 2011; Kipkorir et al., 2022; Mitjà et al., 2022). MPXV can directly infect damaged skin, and replicate in keratinocytes, fibroblasts, and endothelial cells (Mitjà et al., 2022). From the initial infection sites, virus can spread to draining lymph nodes, where the virus can replicate. MPXV can subsequently reach the tonsils, the spleen, and the liver. Replication in these organs results in a second viraemia wave, enabling the virus to access distant organs such as the lung, kidneys, intestines, and skin and causing recognizable clinical manifestation (Giulio and Eckburg, 2004; Damon, 2011; Kipkorir et al., 2022; Mitjà et al., 2022).

Comparison of the Clade I and II strains has provided an indication which viral gene products may be responsible for their difference in virulence. The *D14L* gene which encodes valosin-containing protein (VCP)-MPXV, also known as CCP or MOPICE, is the orthologue of VACV secreted complement C3b/C4b-binding protein VACV-Cop C3L (Chen et al., 2005). This protein is known to inhibit the complement and to contribute to virulence (Kotwal et al., 1990; Isaacs et al., 1992). Compared to the VACV protein, VCP-MPXV/MOPICE is truncated due to a single nucleotide deletion in the *D14L* gene leading to a stop codon (Uvarova and Shchelkunov, 2001). The gene is absent from Clade II strains due to ~10 kbp deletion (Likos et al., 2005). Deletion of the *C3L* gene caused VACV attenuation in infected animal models (Isaacs et al., 1992; DeHaven et al., 2010; Girgis et al., 2011). Studies with Clade I MPXV with deleted *D14L* showed that intranasal infection of prairie dogs resulted in decreased morbidity and mortality. However, ablation of *D14L* did not significantly affect virus replication compared to animals infected with control Clade I virus. On the other hand, Clade II virus with inserted *D14L* gene did not have virulence compared to Clade I virus and no apparent effect on disease-associated mortality compared to control Clade II virus was observed (Hudson et al., 2012). So, other factors besides VCP-MPXV/MOPICE are necessary to explain the difference in virulence between Clade I and II MPXV.

Other candidate virulence genes include *BR-203, BR-209*, and the OPXV major histocompatibility complex class I–like protein orthologue (*OMCP* or *N3R*) (Chen et al., 2005; Likos et al., 2005). BR-203 protein is an orthologue to the myxoma virus M-T4, which plays a role in avoiding apoptosis of infected lymphocytes, hence promoting viral spread within the host (Barry et al., 1997; Weaver and Isaacs, 2008). *Myxoma virus* expressing C-terminal truncated M-T4 caused increased inflammatory response compared to rabbits infected with wildtype virus, whereas challenging with virus lacking M-T4 resulted in disease attenuation (Barry et al., 1997; Hnatiuk et al., 1999). Thus, BR-203 may have a dual function in protecting infected lymphocytes from apoptosis and in modulating the inflammatory response to virus infection.

The *BR-209* gene encodes a 326 aa interleukin-1β (IL-1β) binding protein, which prevents IL-1β from interacting with the IL-1 receptor. Mice intranasally infected with VACV lacking IL-1β binding protein developed a more severe illness than wildtype virus (Alcami and Smith, 1992). Perturbing the IL-1 signaling pathway dampens the innate and acquired immunity, explaining the virulent action of the IL-1β binding protein (Dinarello, 2018). Interestingly, the *BR-209* gene of Clade I strains has two ORFs that can encode a putative N-terminal protein fragment of 210 aa and a C-terminal protein fragment of 126 aa, whereas the Clade II strains encode a putative N-terminal 163 aa polypeptide and a C-terminal 132 aa fragment (Weaver and Isaacs, 2008). It is unknown if any of the fragments function in a way similar to the full-length protein, nor if the differences in the length of the N-terminal fragments of Clade I versus Clade II strains of MPXV contribute to the differences in virulence (Weaver and Isaacs, 2008).

The *OMCP* gene (*N3R*) from Clade I strains isolated in the DRC carries a 628 bp deletion, which removes the 5’region of the *N3R* gene (which encodes the OMCP) and the *N2R* gene (encoding a 73 aa polypeptide with unknown function) (Kugelman et al., 2014). OMPC functions as soluble natural killer group 2, member D receptor (NKG2D) ligand, possibly representing a strategy to avoid NK-cell-mediated killing (Campbell et al., 2007; Lazear et al., 2013). Based on the clinical metadata, it was suggested that the deletion is associated with increased human-to-human transmission and pathogenicity compared to Clade IIa and Clade IIb isolates (Forni et al., 2022a).

The Clade I strain also contains truncated orthologues of VACV-Cop E3L and VACV-Cop K3L, two proteins that function in interferon (IFN) resistance (Weaver and Isaacs, 2008). Clade I and Clade II genomes differ also in *VACV-CopH5R* (the VACV orthologue encodes a late transcription factor, which plays a role in viral replication, transcription and morphogenesis (Van Vliet et al., 2009), *VACV-Cop A9L* (VACV orthologue encodes a morphogenesis factor) (Van Vliet et al., 2009), *VACV-Cop A50R* (VACV orthologue encodes a DNA ligase) (Van Vliet et al., 2009)), and *VACV-Cop A36R* (playing role in actin tail formation) (Wolffe et al., 1998)). The exact mechanisms by which these proteins may also contribute to differences in pathogenicity between Clade I and II strains remains elusive.

### 4.2 Signal transduction and pathways

Manipulation of signaling pathways promotes viral replication and determines disease outcomes, mainly by targeting cell growth and immune responses (Krajcsi and Wold, 1998; Greber, 2002). MPXV infection suppresses expression of host genes whose products are implicated in regulation of histone expression, cytoskeletal rearrangements, cell cycle progression, IFN-associated genes, and signaling pathways such as the nuclear factor kappa B (NFκB), the mitogen-activated protein kinase (MAPK), and metabolic pathways (Alkhalil et al., 2010; Rubins et al., 2011; Xuan et al., 2022).

The NFκB pathway plays pivotal roles in inflammation and immunity (Shao-Cong Sun, 2017). NFκB is typically found as a cytosolic trimer of p65/p50 and the inhibitor protein IκB. Phosphorylation of IκB by the trimer IKKα/IKKβ/IKKγ results in the release and activation of p65/p50, which translocate to the nucleus and act as a transcription factor (Hayden and Ghosh, 2008) (**Figure 4B**). Ankyrin proteins from OPXVs can inhibit the NFκB pathway by interfering with different components of this pathway (Shisler and Jin, 2004; Chen et al., 2008; Mohamed and McFadden, 2009; Ember et al., 2012; Mansur et al., 2013; Herbert et al., 2015). MPXV genome encodes the ankyrin-like proteins J3L, D1L, D7L, D9L, O1L, C1L, B5R, B17R, N4R, and J1R (Shchelkunov et al., 2002; Lum et al., 2022), but their exact function remains to be determined. In addition, the MPXV BCL2-like proteins A47R, B13R, C6R, and P1L can prevent activation of the NFκB pathway (Lum et al., 2022). The MPXV *B1R* gene encodes the VACV Kelch-like protein A55, which inhibits the NFκB pathway and stimulates CD8+ T cell proliferation (Lysakova-Devine et al., 2010). However, it remains to be determined whether B1R exerts a similar function.

The Janus kinase (JAK) and signal transducer and activator of transcription (STAT) pathway mediates cellular responses to cytokines and growth factors (Mythology, 1990). H1L inactivates STAT1, and C6R blocks STAT2 (Mann et al., 2008; Stuart et al., 2016). Whether the MPXV H1L and D11L orthologues act similarly has not been investigated (**Figure 4B**).

The VACV epidermal growth factor homologue VGF (C11R) usurps the epidermal growth factor receptor (EGFR) pathway to provoke cell proliferation and to stimulate efficient virus spread and pathogenesis (Buller et al., 1988; Beerli et al., 2019). The *D3R* gene (OPG019) encodes an EGF homologue that might activate the EGFR pathway and enhance viral dissemination. VACV-Cop F11L also promotes viral spreading by inhibiting the RhoA signaling pathway (Beerli et al., 2019). It is not known whether the MPXV orthologue C17L possesses the same function.

Circumventing apoptosis is used by viruses to achieve productive replication. VACV F1L and N1L proteins can prevent apoptosis by inhibiting pro-apoptotic proteins BAK, BID, BAD, and BAX (Aoyagi et al., 2006; Cooray et al., 2007). It is unknown whether the MPXV C7L and P1L isologues have similar functions. Other MPXV genes that prevent apoptosis include *B12R, B19R, D5R*, and *F3L* (Lum et al., 2022). B12 is a serine protease and its VACV orthologue B13R has been shown to be a caspase inhibitor (Li and Beg, 2000).

Signal transduction is often mediated through a cascade of phosphorylation events (Denhardt, 1996). Kindrachuk et al. compared the phosphorylation pattern of host cell proteins after Clade I or Clade II MPXV infection (Kindrachuk et al., 2012). They found that Clade I MPXV infection down-regulated pathways related to cell proliferation and apoptosis as compared with Clade II MPXV. Differences in MPXV-induced posttranslational modification may explain the differences in virulence between MPXV clades.

### 4.3 Host immune response and virus immune decoy mechanisms

#### 4.3.1 Host immune response

Human infection with MPXV is associated with increased levels of ILs, C-C motif chemokine ligand 2 (CCL2) and CCL5 (Johnston et al., 2015), and a significant decrease in tumor necrosis factor alpha (TNF-α), IFN-γ, and IL-2 and IL-12 (Li et al., 2022). MPXV infection provokes IgM and IgG antibodies, long-term persistence of residual IgG-memory B cells, and a rapid expansion of activated effector CD4+ and CD8+ T cells followed by a decrease over time (Agrati et al., 2022; Mitjà et al., 2022). MPXV also interferes with adaptive immune responses of antiviral CD8 + and CD4 + T cell responses via inhibiting T cell receptor-mediated T cell activation (Hammarlund et al., 2008).

#### 4.3.2 Evasion host immune defense

Because of the high homology between the MPXV genes and the corresponding OPXV orthologues, it is assumed and, in some cases, demonstrated that MPXV applies similar strategies to evade the host immune defense system. The Toll-like receptor (TLR) family functions as pattern recognition receptors, which recognize damage-associated molecular patterns such as viral dsRNA. Binding of viral dsRNA to specific TLR members triggers the expression of proinflammatory molecules involved in host anti-viral responses and subsequent activation of the adaptive immune defense system (Mogensen, 2009). The VAVC A46 protein inhibits the TLR4 signaling pathway (Lysakova-Devine et al., 2010). However, it is unknown whether the MPXV orthologue A47 has the same property, but the A47 protein has structural similarities to VACV protein A52R, which can inhibit the TLR3 and TLR4 signaling pathways (Bowie et al., 2000; Harte et al., 2003), underscoring a role for MPXV A47 in interfering with TLR signaling. MPXV produces low levels of dsRNA intermediates (Arndt et al., 2016), but whether these are recognized by TLR3 has not been investigated, although transcriptome analysis of MPXV-infected cells revealed repression of TLR3 target genes (Rubins et al., 2011). dsRNA can also activate kinase R (PKR), which mediates phosphorylation of eIF2α, resulting in the inhibition of viral and cellular mRNA translation (Pears, 1995). VACV E3 and K3 are inhibitors of PKR, allowing VACV to evade an antiviral response (Seet et al., 2003; Deng et al., 2008). The N-terminal domain of VACV E3 protein is absolutely required for the interaction with dsRNA (White and Jacobs, 2012). MPXV F3 protein is the VACV E3 protein homologue with a 37 aa truncation at the amino terminus (Arndt et al., 2015), suggesting that MPXV F3 does not bind dsRNA. However, MPXV can inhibit host immune responses, although a recombinant VACV expressing the MPXV *F3L* gene did not inhibit host PKR activation (Arndt et al., 2015), suggesting that MPXV has evolved to encode for yet undiscovered proteins that compensate for the missing N-terminal amino acids of F3 in limiting host antiviral activities. As previously mentioned, MPXV CCP (*D14L* gene) is completely absent in Clade II isolates, whereas the Clade I strains are predicted to express CCP, albeit with a truncated fourth short consensus repeat (Yeh and Contreras, 2022). Loss of expression of CCP/MOPICE limited the adaptive immune response against MPXV infection in rhesus macaques (Estep et al., 2011).

IFNs are main effectors of the innate immune response and can inhibit virus replication (Seet et al., 2003; Randall and Goodbourn, 2008). The MPXV B16 protein (VACV B19 orthologue) is a secreted type I IFN inhibitor and suppresses the antiviral type I IFN-induced signaling pathway (Fernández de Marco et al., 2010). VACV K7 abrogates IFN signaling by destabilizing IFN-regulated factor 3 (IRF3) and inhibits NFκB activation, whereas VACV H1 can block IFN signaling (Benfield et al., 2013; Liu and Moss, 2018). Whether the MPXV D9 and H1 orthologues have the same function remains to be proven, but both proteins are predicted to interact with several cellular proteins of the immune system (Mann et al., 2008; Kumar et al., 2023). VACV E3 perturbs IFN signaling by binding host Z-DNA binding protein 1 (ZBP1). The MPXV E3 orthologue (F3) perturbs the IFN pathway, but whether in a ZBP1-dependent manner needs to be established. The DNA-sensing receptor pathway cGAS/STING (cyclic GMP-AMP synthase/stimulator of interferon genes) can activate the IFN and NFκB pathways (Motwani et al., 2019). The VACV B16 and B8 proteins block IFN signaling by operating as soluble IFN-α and IFN-γ receptors, respectively (Alcamí and Smith, 1995; Colamonici et al., 1995). The MPXV ORF B16R and B9R encode functional homologues (Shchelkunov et al., 2002). OPXVs encode poxins that degrade 2′,3′ cGAMP and thereby inhibit cGAS/STING signaling (Eaglesham et al., 2019). Poxin is conserved in MPXV where it is fused to an additional C-terminal domain previously noted to have homology with human schlafen proteins (VACV B2R = MPXV B4R) (Eaglesham et al., 2019), hence MPXV may evade the immune system by targeting the cGAS/STING pathway.

MPXV can dodge the immune system by targeting the antiviral cytokine TNFα and other immunomodulating molecules. The MPXV-encoded cytokine response-modifying protein B (CrmB; J2L or OPG002) functions as a decoy receptor for TNFα (Gileva et al., 2006). The C-terminal domain of VACV CrmB can bind CCL28, CCL25, CXC motif chemokine ligand 12 (CXCL12), CXCL13 and CXCL14 (Alejo et al., 2006). It is unknown whether MPXV CrmB interacts with these chemokines, but the amino acid sequence of the corresponding domain in MPXV CrmB differs significantly (Gileva et al., 2006).

Natural killer (NK) cells and cytotoxic T cells (CTL) play a crucial role in eliminating viral infections. Although the number of NK cells expand significantly in peripheral blood and lymph nodes in MPXV-infected rhesus macaques, their migrating capacity was reduced, and several functions such as expression of chemokine receptors (including CCR5, CCR6 and CXCR3) and secretion of IFNγ and TNFα were impaired (Song et al., 2013). The importance of NK cells in controlling MPXV viral load was demonstrated in the highly vulnerable to OPXV infection CAST/EiJ mouse strain owing to low numbers of NK cells. IL-15 treatment, known to increase the numbers of IFNγ-secreting NK cells and CD8+ T cells, protected CAST/EiJ mice from lethal MPXV infection even when both CD4+ and CD8+ T cells were depleted. This implies that the expanded NK cells were responsible for the protective effect (Al-musa et al., 2022; Lum et al., 2022).

MPXV protein B10 avoids detection of virus-infected cells by CTL by impairing peptide loading and MHC-I trafficking within the endoplasmic reticulum. However, NK cells continually screen cells via NKG2D for the absence of MHC-I, thereby ensuring that the MHC system is not compromised (Lum et al., 2022). MPXV-infected cells with downregulated MHC-I expression overcomes detection by NK cells by secreting the OCMP protein that binds NKG2D and suppress the typical NKG2D-dependent NK cell lysis of cells that do not express MHC-I (Lum et al., 2022) (**Figure 4C**). IL-18, an IFNγ-inducing factor, stimulates the synthesis of various cytokines and chemokines, regulates Th1 and Th2 cell responses, and activates NK and CTL (Seet et al., 2003). OPXVs block IL-18 activities by producing an IL-18 binding protein (Born et al., 2000). The MPXV *D6L* gene encodes such IL-18 binding protein (Shchelkunov et al., 2002).

The MPXV *J3R* and *A41L* genes encode chemokine binding proteins (Shchelkunov et al., 2002), which are assumed to destroy the chemokine concentration gradient resulting in decreased neutrophil migration in tissues infected with MPXV and thus reducing viral virulence and inflammatory response (Bahar et al., 2008).

The complement system, which forms an essential part of the innate immune system is activated early in MPXV infection in mice, and is crucial for viral control (Moulton et al., 2008). MPXV MOPICE which from studies with VARV and VACV MOPICE was found to inhibit activation of the complement pathway (Liszewski et al., 2006). MOPICE modulates the antiviral immune response as observed by enhanced viral replication *in vivo* and dampened adaptive immune response in rhesus macaques infected with MPXV lacking MOPICE expression (Estep et al., 2011; Hudson et al., 2012). Because of the absence of the *D14L* gene in the Clade II, the virus and virus-infected cells would be predicted to be susceptible to the host complement attack. However, the expression of MOPICE in Clade II did not increase its virulence (Hudson et al., 2012), demonstrating that MOPICE is not the sole determinant of differences in viral virulence between the two clades.

Less is known how MPXV may interfere with the adaptive immune system. MPXV interferes with adaptive immune responses of antiviral CD8 + and CD4 + T cell responses via inhibiting T cell receptor-mediated T cell activation (Hammarlund et al., 2008).VACV A35 blocks immune priming of T lymphocytes by interfering with MHC class II-restricted antigen presentation. Moreover, infection studies in cells with MPXV lacking the *A35R* gene demonstrated that A35 inhibits production of cytokines and chemokines (Rehm et al., 2011). MPXV A35 analogue, A37, might suppress presentation of viral antigens to immune cells and help the virus to evade the host immune defense system. However, MPXV does not seem to interfere with MHC expression or intracellular transport of MHC molecules (Hammarlund et al., 2008).

## 5 Epidemiology

### 5.1 Demographic and epidemiological characteristics

**Figure 5:**
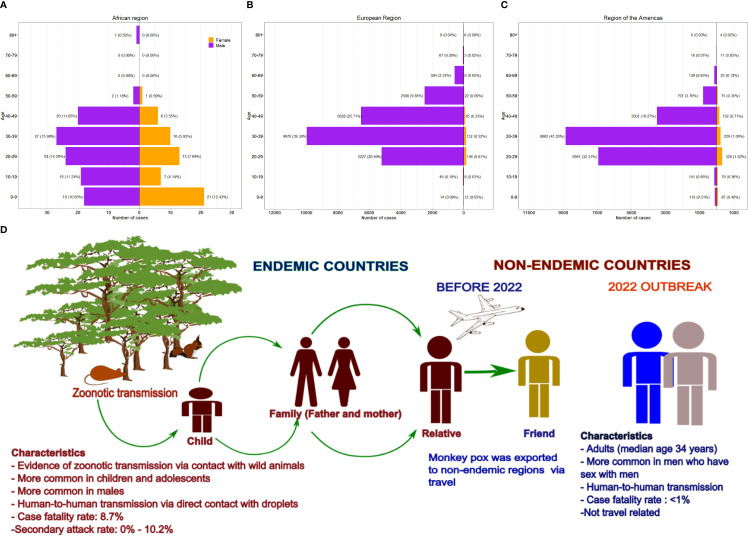
Demographic characteristics and transmission routes of human mpox. Demographic characteristics of 2022 human mpox cases according to sex and age in three WHO regions based on data from 4727328. **(A)** African region, **(B)** European region and **(C)** region of Americas. The pyramid plots show the number of cases and percentage of overall cases. **(D)**Transmission of mpox in endemic and non-endemic regions pre-2022 and 2022 outbreak. The characteristics of outbreaks in the different regions were highlighted. Prior to 2022, mpox was limited to the endemic regions and cases outside the region were usually travel related. However, the 2022 outbreak in non-endemic region was not travel related and has been reported in several countries.

Surveillance data on mpox in endemic countries during different periods between 1970-2015 showed that 71%-83% of the disease occurred in children (<10 years of age) and 51%-67% in males (Breman et al., 1980; Heymann et al., 1998; Rimoin et al., 2011). In contrast, the median age for the 2017-2018 outbreak in Nigeria was 29 years, with males accounting for 64% of the cases (Yinka-Ogunleye et al., 2019). However, for the 2022 multi-country outbreak, males accounted for 96.8% of the cases, and the median age was 34 years (Interquartile range: 29 – 41) (World Health Organization., 2022). In the African region, children (0-9 years of age) accounted for 23.08% of mpox cases compared to <1% in Europe and the Americas. Notably, the male-to-female ratio in Africa is also markedly lower than in other regions (**Figures 5A-C**) (World Health Organization, 2022).

### 5.2 Case fatality rate

Before the 2022 outbreak, the overall pooled estimated case fatality rate (CFR) was 8.7% and varied by the clade of the virus (Bunge et al., 2022). The pooled CFR for Clade II was 3.6% and 10.6% for Clade I (Bunge et al., 2022). The 2022 outbreak has an estimated CFR of 0.08% (World Health Organization, 2022). The low CFR in the current outbreak may be related to several factors, including the fact that the Clade II has a CFR of <1%, active surveillance, early diagnosis, and treatment (World Health Organization, 2022b).

### 5.3 Secondary attack rate

Over the past five decades, the secondary attack rate for mpox has been stable and ranges from 0% to 10.2% (Breman et al., 1977; Merouze and Lesoin, 1983; Tchokoteu et al., 1991; McCollum et al., 2014; Besombes et al., 2019; Ye et al., 2019). Higher estimates were reported in the 2013 outbreak in the DRC (50% among 16 households) and Nigeria (71% in the 2017- 2018 outbreak from a single household) (Nolen et al., 2015; Beer and Bhargavi Rao, 2019; Bunge et al., 2022). However, it is imperative to note that it is unclear how the high estimates were obtained and thus may likely be overestimated (Beer and Bhargavi Rao, 2019).

### 5.4 Virus reproduction number

Reproduction number (R_0_) is the number of secondary cases anticipated to develop from a single primary case in a naive population (Beer and Bhargavi Rao, 2019). While historical data on R_0_ is limited, published evidence from DRC’s active surveillance between 1980 and 1984 estimated R_0_ to be 0.8 (Beer and Bhargavi Rao, 2019), implying that transmission is ineffective as a human-to-human epidemic is likely to die out (Beer and Bhargavi Rao, 2019). However, early estimations of the R_0_ in the 2022 outbreak in non-endemic countries showed a high variability ranging from 1.54 (Belgium) to 3.62 (Germany), with a median of 2.44 (Branda et al., 2023). This suggests sustained human-to-human transmission, possibly due to several contributing factors, including different social and/or sexual behaviors, different MPXV variants, population density or other unknown causes (Branda et al., 2023). While the early estimations indicated sustained transmission, the outbreak has slowed down with case reductions. This could be due to behavior change, infection induced immunity, and vaccinations.

### 5.5 Risk factors (sexual and social networks, smallpox vaccination) and co-morbidities

Transmission of MPXV can occur from animal-to-human (zoonotic) and from human-to-human (interhuman). Zoonotic transmission usually happens through contact with an infected animal’s bodily fluid or through a bite or scratch (Reynolds et al., 2007). Exposure to animal reservoirs, especially in regions with deforestation enhancing animal-human contact, and uncooked meat products are major risk factors for zoonotic transmission (Kipkorir et al., 2022). During the 2003 USA outbreak, exposure was classified as “non-invasive” (touching an infected animal) or “complex” (invasive bite from an ill animal; non-invasive exposure i.e., any exposure that did not break the skin) (Reynolds et al., 2018). Patients with complex exposures were more likely to develop systemic illness compared to those with non-invasive exposure (Reynolds et al., 2018). Dewitt et al. predicted the MPXV mode of transmission in most 2022 studies to be caused by inter-human transmission (Dewitt et al., 2022) (**Figure 5D**). Large respiratory droplets, bodily fluids, contaminated fomites, and viral shedding through feces are also considered risk factors for viral transmission (Rimoin et al., 2010; Sklenovská and Van Ranst, 2018; El Eid et al., 2022). Airborne transmission of MPXV between animals in experimental settings has also been reported and MPXV was detected in upper respiratory samples, suggesting that interhuman transmission of MPXV via the airborne route may be possible. However, epidemiological observations do not support airborne transmission as the primary route of transmission (Riopelle et al., 2022). The virus can also cross the placenta, suggesting vertical transmission (Mbala et al., 2017; Cuerel et al., 2022). At least 12 pregnant women have been infected during the 2022 outbreak, but vertical transmission was not observed in any case (Khalil et al., 2020).

MPXV has also been detected in human semen (Antinori et al., 2022; Noe et al., 2022), and in archival testes tissue of crab-eating macaque (Liu et al., 2022), suggesting potential sexual transmission of the virus. Recent outbreak suggests that MSM subpopulation may also be at an increased risk (Kipkorir et al., 2022).

In addition, waning immunity against smallpox has been considered another potential risk factor for the disease (Rimoin et al., 2010). Evidence from early outbreaks in the 1980s showed that previous smallpox vaccination provided 85% protection against mpox (Rimoin et al., 2010). Most cases reported were among those born after vaccination ceased in 1980, and herd immunity has significantly decreased (Rimoin et al., 2010).

Evidence from the literature suggests that those who are immunocompromised due to HIV and other underlying conditions are at increased risk of severe mpox disease (Center for Diseases Control and Prevention (CDC), 2022b) (Yinka-Ogunleye et al., 2019). A high proportion of patients with mpox in the 2022 outbreak had concurrent HIV infection and sexually transmitted infections (STI) (Fischer et al., 2022; Ghaffar and Shahnoor, 2022). Mpox patients with HIV infection were more likely to be hospitalized than those without HIV infection (Curran et al., 2022). However, the evidence on the reason for hospitalization is limited, and it is unknown if it reflects a more severe mpox illness (Curran et al., 2022).

Furthermore, co-infection with other conditions with rashes, such as a varicella-zoster virus (VZV), can occur (Hughes et al., 2021; Stephen et al., 2022). This herpesvirus causes chickenpox and shingles and is frequently misdiagnosed as mpox in regions where both diseases are endemic (Hoff et al., 2017; Hughes et al., 2021). Chickenpox is exclusive to humans and more common in younger age groups, and co-infection with mpox has been reported more commonly among children (Leung et al., 2019; Hughes et al., 2021).

## 6 Diagnosis, screening, prevention, and treatment

### 6.1 Diagnosis and case definitions

Electron microscopy, immunohistochemical detection of MPXV proteins, and detectable levels of anti-OPXV IgM antibody during the period of 4 to 56 days after rash onset can be used to diagnose MPXV infection, but all methods are not specific (Center for Diseases Control and Prevention (CDC), 2022a). According to the Centers for Disease Control and Prevention (CDC) (Center for Diseases Control and Prevention (CDC), 2022a), mpox cases should be confirmed by real-time polymerase chain reaction (qPCR) or Next-Generation sequencing and isolation of MPXV in culture from a clinical specimen. The *F3L*, *E9L*, *B6R* and *J2R* genes are all target of qPCR in MPXV diagnosis (Li et al., 2010; Alakunle et al., 2020; Altindis et al., 2022; Cheema et al., 2022; Nakhaie et al., 2022). The suspected mpox cases are characterized by fulfilling one of the epidemiological criteria (within 21 days of illness onset), as outlined by CDC (Center for Diseases Control and Prevention (CDC), 2022a).

### 6.2 Surveillance and contact tracing

One of the crucial ways of controlling the spread of mpox is contact tracing (Harapan et al., 2022; Kalyar et al., 2022). Individuals exposed to MPXV should be monitored for 21 days checking mpox symptoms, and those with suspected or confirmed mpox cases should be isolated to avoid infecting others (Titanji et al., 2022). Velavan et al. predicted the mpox outbreak would not last provided that cases are well isolated through the contact tracing (Velavan and Meyer, 2022). Although much attention is given to human-human contact tracing, great efforts need to be put into animal-animal and animal-human contact tracing especially due to non-specificity of reservoir hosts for MPXV (Petersen et al., 2019a). Surveillance cannot be undermined in curtailing mpox as surveillance would provide more insight into the epidemiology of the disease (Riopelle et al., 2022). In Nigeria, the Outbreak Response Management and Analysis System (SORMAS) for mpox surveillance across portions of 8 states was implemented in November 2017 for the mpox outbreak. The use of the system increased the quantity of epidemiological data collected and the communication of aggregate case data (Mauldin et al., 2022).

### 6.3 Vaccine

Although there is no specific vaccine for MPXV (Hobson et al., 2021; Sah et al., 2022a), the smallpox vaccines have been reported to give 85% cross-immunity against MPXV due to shared antigenic features (Alakunle et al., 2020).

ACAM2000™ is a replication-competent vaccinia virus vaccine licensed by Food and Drug Administration (FDA) in August 2007 for smallpox prevention, and it is derived from a single clonal viral isolate from Dryvax (Gruber, 2022; Poland et al., 2022) which is a first-generation smallpox vaccine. As a second-generation attenuated vaccinia virus vaccine (Gessain et al., 2022), ACAM2000™ has been recommended as MPXV post-exposure prophylaxis (Aden et al., 2022; McCarthy, 2022). Although high level of protection against mpox in animal models has been recorded, the safety of ACAM2000™ in humans is still of great concern as cardiac complications, and extremely painful and uncomfortable cutaneous reaction at the injection site have been associated with the vaccine (Chandran et al., 2022). Therefore, the vaccine is no longer licensed by European Union (Luo and Han, 2022).

JYNNEOS (Imvamune or Imvanex) was approved by FDA in September 2019 for prevention of smallpox and mpox in adults aged >18years (Alakunle et al., 2020). JYNNEOS is the brand name of Modified Vaccinia virus Ankara Bavarian Nordic (MVA-BN) vaccine (Kmiec and Kirchhoff, 2022), a non-replicating third-generation attenuated vaccine. JYNNEOS is considered safer (with proven efficacy in animals and humans) than ACAM2000™ (Sah et al., 2022a). The Advisory Committee on Immunization Practices has recommended JYNNEOS as an alternative to ACAM2000™ (Harapan et al., 2022). Nonetheless, both vaccines (JYNNEOS and ACAM2000™) have been recommended for MPXV high-risk groups (Petersen et al., 2022).

LC16m8 is another potential vaccine for MPXV which is obtained by subjecting VACV lister to 36 serial passages at low temperature (30°C) in primary rabbit kidney cells (Domán et al., 2022; Poland et al., 2022; Schnierle, 2022). As a third-generation attenuated vaccine (Gessain et al., 2022), LC16m8 has been shown to be protective against MPXV in animal models with lower neurotoxicity (Domán et al., 2022; Schnierle, 2022). The frameshift mutation in LC16m8’s major extracellular enveloped virion antigen (B5R) contributes to the vaccine replication competence and low virulence (Domán et al., 2022). Presently, LC16m8 is only licensed in Japan (Domán et al., 2022; Schnierle, 2022).

### 6.4 Antivirals

Although there are no specific antivirals for mpox, some antivirals (tecovirimat, brincidofovir, cidofovir) have been explored (Riopelle et al., 2022). Tecovirimat (ST-246 or TPOXX^®^), 4-trifluoromethylphenol derivative, was approved (for smallpox) by FDA in 2018 and approved by European Medicines Agency in January 2022 for treatment of smallpox and cowpox. Tecovirimat inhibits VP37 (p37) protein of VACV by targeting the viral *F13L* gene (Frenois-Veyrat et al., 2022) and the CPXV homolog *V016* gene (Siegrist and Sassine, 2022). VP37, a highly conserved protein in OPXV genus, is required for viral maturation and release from the infected cell. Inhibition of VP37 prevents viral spread within an infected animal models (Rizk et al., 2022). Goyal *etal*. recommended tecovirimat to be administered as first line of mpox treatment in pregnant and breastfeeding patients (Goyal et al., 2022). A tecovirimat analogue (synthesized by the State Research Center of Virology and Biotechnology, Russia) has been highlighted as a promising antivirals against OPXV infections (Singhal et al., 2022).

Cidofovir (CDV or Vistide^®^) prodrug is an acyclic nucleoside phosphate (Alakunle et al., 2020) that was approved by FDA in 1996 for the treatment of retinitis (caused by cytomegalovirus) in AIDS patients (Harapan et al., 2022). The efficacy of CDV has been identified during the *in vitro* studies in MPXV-infected animals, but the clinical data of CDV efficacy against mpox in human are not available. Brincidofovir (CMX001 or hexadecyloxypropyl-cidofovir), a CDV derivative, was approved for smallpox treatment in 2021 by FDA, and it has lesser toxic effects than CDV (Ortiz-Saavedra et al., 2022). Evaluation of CMX001 efficacy and safety in human mpox through the clinical trials is needed (Ortiz-Saavedra et al., 2022; Siegrist and Sassine, 2022).

## 7 Biosafety, Biosecurity and Bioethics

### 7.1 Recombination with vaccinia virus and other OPXV

Due to inadequate genome surveillance data particularly in endemic regions, little information about MPXV recombination is available (Yeh et al., 2022). However, there are some evidences of recombination between coinfecting or superinfecting OPXVs both in a laboratory setting and in nature (Estep et al., 2011; Goff et al., 2011; Brennan et al., 2022; Diaz-Cánova et al., 2022b, 2022a; Gigante et al., 2022).

MPXV circulating in non-endemic regions where other OPXVs are endemic, for instance, CPXV in Europe and VACV-like in south America, and vaccination against mpox with JYNNEOUS or ACAM 2000 are scenarios for coinfection and superinfection between different species of OPXV. Thus, there remains a potential risk of recombination between MPXV and other OPXVs that may result in MPXVs with mosaic genomes and altered biological characteristics.

### 7.2 Dual use and bioterrorism

Although there is insufficient evidence of MPXV being used for bioterrorism at the moment (Hosseini-Shokouh et al., 2022), many scientists have expressed concerns over its potential use for bioterrorism because there is a report that there was an attempt by the former Soviet Union to use MPXV as a bioweapon (Nalca et al., 2005; Duraffour et al., 2007; Kindrachuk et al., 2012; Hosseini-Shokouh et al., 2022; Makkar, 2022). The possibility of MPXV as a potential bioweapon due to its global reemergence and its clinical similarities with VARV has placed the virus on the global public health agenda (Ellis et al., 2012; Ihekweazu et al., 2020). For example, the USA has made preparation for the possibility of smallpox virus as a potential bioterror biological by storing smallpox vaccines and antivirals after the 9/11 attack (Franz, 2004; Xiang and White, 2022). Smallpox virus is in category A of the CDC list of bioterrorism agents (Hosseini-Shokouh et al., 2022). The risk of bioterrorism is heightened knowing that MPXV genomes can be synthesized from publicly available sequence data and life viruses (with or without further genetic modification) can be re-constituted. This has already been demonstrated with horsepox virus (HPXV), which shares the same genus as MPXV (Noyce et al., 2018). Hence, a strict dual-use policy must be agreed upon and implemented in all laboratories across the globe.

### 7.3 Stigmatization and vaccine inequity

Sexual orientation (especially LGBQTI+ community) (Sousa et al., 2022) and racial (Happi et al., 2022) are two stigmas associated with MPXV. The persistent narrative of the media alongside many scientists linking the mpox 2022 outbreak to Africa/West Africa/Nigeria is worrisome (Sousa et al., 2022). Despite the mpox 2022 outbreak, which occurred outside Africa, the nomenclature and the geographical labels of MPXV strains still reference West African, even though the origin of this outbreak is still unresolved. Furthermore, high mpox cases were reported among MSM (Happi et al., 2022; Malta et al., 2022; Sousa et al., 2022; Sah et al., 2022b). This narrative portrays these men contracting MPXV because they engaged in sexual intercourse with fellow men, meanwhile the spread of the virus can occur regardless the sex. Vaccine inequality affects lower- and middle-income countries (LMICs). An unknown number of mpox cases in LMICs are not captured due to a shortage of resources like limited testing and surveillance capacity (Malta et al., 2022). As highlighted by Malta et al., the MPXV vaccines are presently accessible only in high-income countries (Canada, the USA, and the UK) (Malta et al., 2022).

## 8 Conclusion

MPXV has emerged and re-emerged for over five decades and yet not much is known about its virological profile and the characteristics of the disease it causes. In particular, the reservoir host of MPXV remains unknown, the viral, host and environmental factors that modulate the virus maintenance in the wild, animal-to-animal transmission, zoonotic transmission and reverse spillover are still a mystery. Neither are we closer to reliable prognostication of virus emergence and accurate modelling of the disease outcomes. Current and future studies should prioritize understanding the molecular basis of MPXV infection to develop effective drugs and vaccines against mpox as well as functional mutational studies that will shed insight into the dynamics of MPXV transmission across hosts. To improve tracking of MPXV, laboratories particularly in resource-poor countries should be equipped with genome-based surveillance capacity and capability. Lastly, MPXV and mpox affect human, animal, and ecosystem health. Thus, a One Health strategy is indispensable to the prevention and treatment of current and future outbreaks.

## Author contributions

MO: Conceptualization, Data curation, Investigation, Methodology, Supervision, Writing – original draft, Writing – review & editing. EA: Formal analysis, Investigation, Methodology, Writing – original draft, Writing – review & editing. DK: Formal analysis, Methodology, Writing – original draft. DD: Data curation, Formal analysis, Investigation, Writing – original draft. FA: Investigation, Writing – original draft. OA: Formal analysis, Investigation, Writing – original draft. UM: Conceptualization, Investigation, Methodology, Writing – original draft, Writing – review & editing.
